# A constraint-based model of *Scheffersomyces stipitis* for improved ethanol production

**DOI:** 10.1186/1754-6834-5-72

**Published:** 2012-09-21

**Authors:** Ting Liu, Wei Zou, Liming Liu, Jian Chen

**Affiliations:** 1 State Key Laboratory of Food Science and Technology, Jiangnan University, 1800 Lihu Avenue, Wuxi, Jiangsu, 214122, China; 2 Key Laboratory of Industrial Biotechnology, Ministry of Education, Jiangnan University, 1800 Lihu Avenue, Wuxi, Jiangsu, 214122, China

**Keywords:** Scheffersomyces stipitis, Genome-scale metabolic model, Constraint-based simulation, Xylose utilization, Ethanol production

## Abstract

**Background:**

As one of the best xylose utilization microorganisms, *Scheffersomyces stipitis* exhibits great potential for the efficient lignocellulosic biomass fermentation. Therefore, a comprehensive understanding of its unique physiological and metabolic characteristics is required to further improve its performance on cellulosic ethanol production.

**Results:**

A constraint-based genome-scale metabolic model for *S. stipitis* CBS 6054 was developed on the basis of its genomic, transcriptomic and literature information. The model *i*TL885 consists of 885 genes, 870 metabolites, and 1240 reactions. During the reconstruction process, 36 putative sugar transporters were reannotated and the metabolisms of 7 sugars were illuminated. Essentiality study was conducted to predict essential genes on different growth media. Key factors affecting cell growth and ethanol formation were investigated by the use of constraint-based analysis. Furthermore, the uptake systems and metabolic routes of xylose were elucidated, and the optimization strategies for the overproduction of ethanol were proposed from both genetic and environmental perspectives.

**Conclusions:**

Systems biology modelling has proven to be a powerful tool for targeting metabolic changes. Thus, this systematic investigation of the metabolism of *S. stipitis* could be used as a starting point for future experiment designs aimed at identifying the metabolic bottlenecks of this important yeast.

## Background

Along with the increasing stress on the shortage of oil reserves and the negative ecological impacts of greenhouse gas emissions, there is a trend for searching of the renewable clean fuels to substitute the traditional fossil fuels worldwide [1-3]. Currently, bioethanol produced from lignocellulosic biomass (second generation bioethanol) has been widely recognized as one of the most attractive alternatives [4]. However, owing to the complex components and rigid structure of plant biomass [5], it is particularly vital to get a robust industrial strain for the efficient bioconversion of lignocellulosic sugars to ethanol [6]. However, none of the screened or engineered strain has been capable of highly efficient bioethanol production from lignocellulosic biomass yet [7]. Thus the searching of an optimal microbial host is still in process. Among the diverse exploited ethanol producers, *Saccharomyces cerevisiae* is considered as the most suitable biocatalyst for industrial ethanol production from sugars or starch feedstocks for its well-characterized genetics, ample genetic tools, high ethanol productivity and so on [8]. Another intensively studied microorganism possessing several appealing characteristics for ethanol production is *Zymomonas mobilis*. It was reported that for *Z. mobilis* the maximum yield of ethanol could reach 97% of the theoretical yield and the tolerance of ethanol was up to 120 g/l [9]. However, an unnegligible drawback of the above-mentioned ethanologenic microbes is that they cannot naturally ferment pentose sugars, the main components of hemicellulose. Although many metabolic engineering strategies, typically the introduction of xylose metabolic pathway to *Z. mobilis* and *S. cerevisiae*, have been carried out to develop more efficient ethanol producers, the success is not very satisfactory [10,11].

A naturally occurring xylose-fermenting yeast *Scheffersomyces stipitis*, formerly known as *Pichia stiptis*[12], was proposed as one of the potential cellulosic bioethanol strain. The most dominant feature of this unconventional yeast is that it’s capable of catabolizing glucose, mannose, galactose, rhamnose, xylose, arabinose, cellobiose, and even some lignin-related compounds [13]. Other advantages include high production capability with a maximum ethanol yield of 0.48 g/g xylose [14], simple growth requirements, strong resistance to contamination and detoxification of the biomass-derived inhibitors [15]. However, some metabolic mechanisms involved in the production of bioethanol in *S. stipitis* were unclear, such as the slow sugar consumption rate [16] and the tough control of precise oxygenation [17]. Besides, the physiological and genetic features of *S. stipitis* were poorly characterized, which hinders the effective gene manipulation for strain improvement. Hence, a systematic understanding of physiological features and metabolic capacities of *S. stipitis* is in great need and genome-scale metabolic model (GSMM) could provide such a platform.

Up to now, there are more than 80 published genome-scale metabolic models (http://systemsbiology.ucsd.edu/InSilicoOrganisms/OtherOrganisms) and the number is still growing owing to the high-throughput genome sequence technologies. GSMMs have been successfully applied to many aspects, such as the design of the metabolic engineering strategies, the understanding of microbial physiology, the contextualization of various omics data, etc. [18,19]. Recently, A GSMM for *S. stipitis* has been reconstructed to investigate some key metabolic traits [20]. Using a different approach, a new constraint-based model *i*TL885 is presented. Compared with the previous model, our model captured more metabolic genes for the adoption of an integrated genome annotation way. In addition, many carbohydrate metabolic pathways were included to represent the unique characteristic of *S. stipitis*. Aside from the study of the physiological changes of ethanol production, the new model was mainly used to make predictions in prior to experiment validation, which is one of the most important applications of constraint-based models. In this research, the proposed model was used to predict the essentiality of the genes and evaluate the capacity of ethanol production with xylose as carbon source.

## Results and discussion

### Reconstruction and description of model *i*TL885

**Figure 1:**
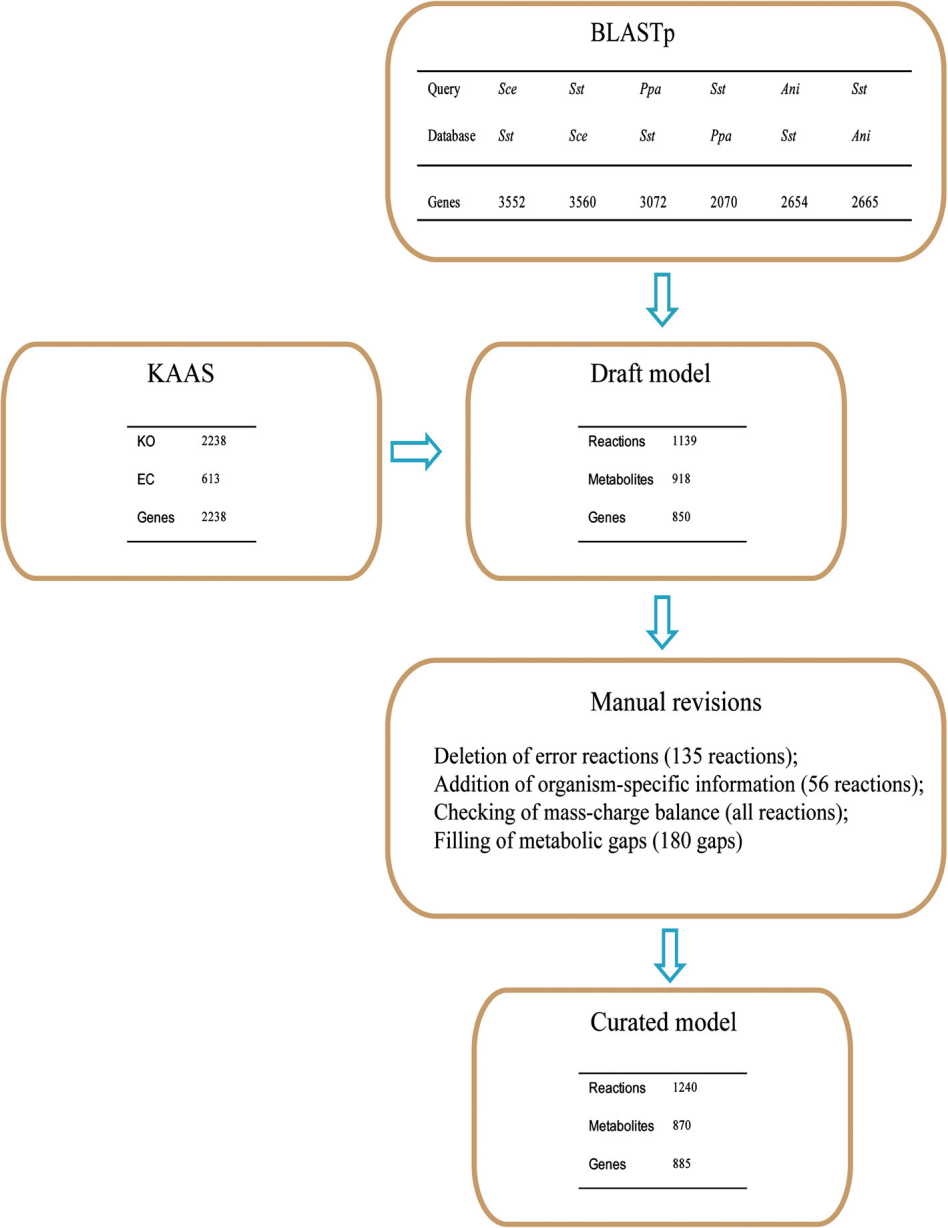
**Procedure of the model reconstruction.** In BLASTp, *Sst*, *Sce, Ppa*, and *Ani* refer to *S. stipitis*, *S. cerevisiae*, *P. pastoris*, and *A. niger* respectively, and genes mean the matched genes between the two genome.

Genome-scale metabolic network was reconstructed using an automated procedure in combination with the manual refinement (see Methods section). Following the main reconstruction steps described in Figure 1, functional annotation of the whole genome was firstly performed by two different sequence similarity search programs. All the matched genes of *S. stipitis* were retrieved from the genome of *S. cerevisiae*, *Pichia pastoris*, and *Aspergillus niger* respectively by the Basic Local Alignment Search Tool (BLAST). Meanwhile, a ‘KO list.xlsx’ with KEGG ORTHOLOGY (KO) identifiers corresponding to the assigned genes was obtained by KEGG Automatic Annotation Server (KAAS). By the integration of the two genome annotation results, a draft model including 1139 reactions and 850 genes was achieved. Then, the draft model was curated with the biochemical information from databases and literature (Figure 1). Detailed genome annotation result was provided in Additional file 1.

**Figure 2:**
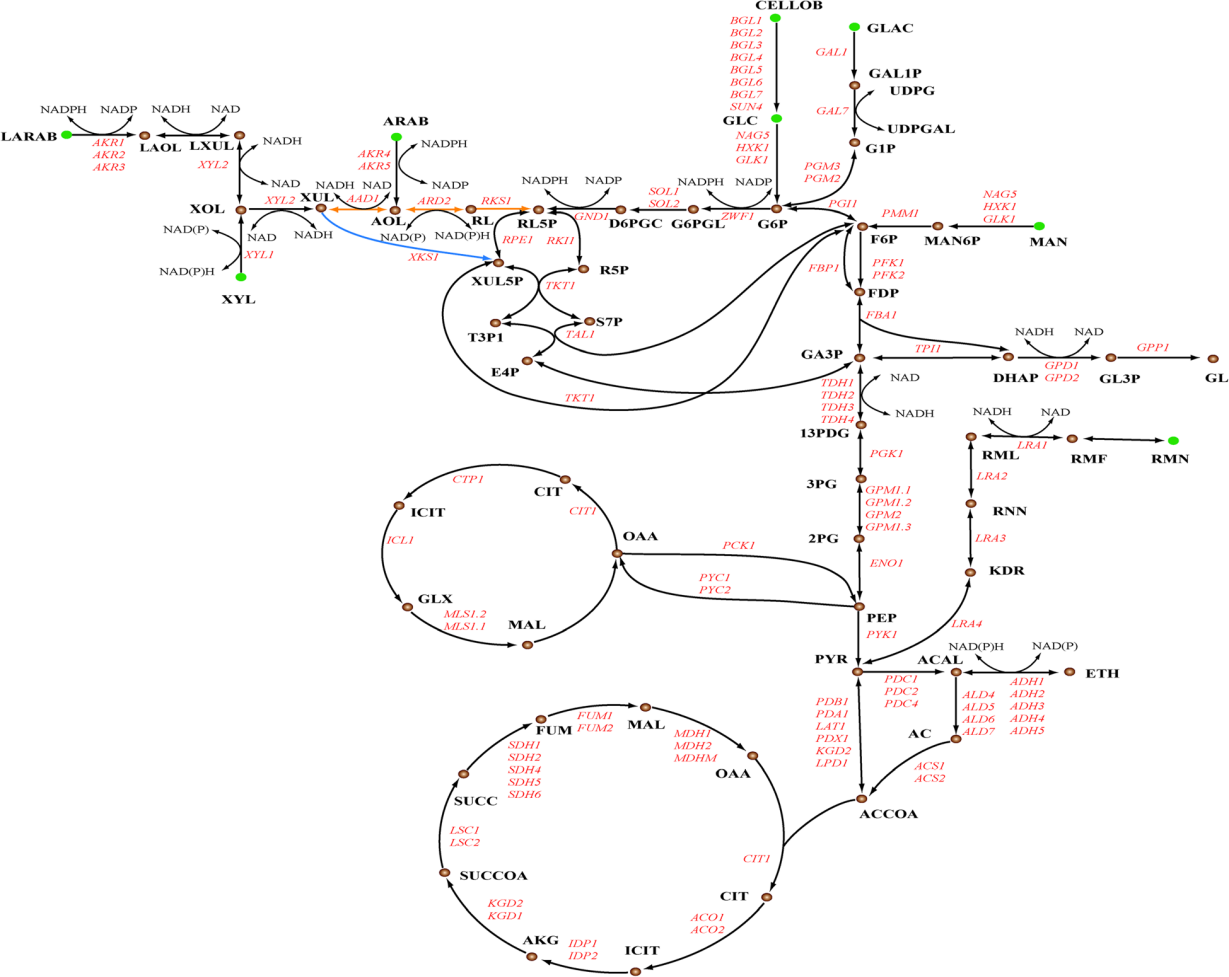
**Schematic illustration of metabolic pathways of cellobiose, rhamnose and five lignocellulose-derived sugars (glucose, mannose, galactose, xylose and arabinose).** The abbreviations of the metabolites and genes are available in Additional file 2 .

During model reconstruction, various sugar transport reactions and metabolic pathways were manually checked and added to the draft model for a better understanding of the carbohydrate metabolism in *S. stipitis*. By the combination of information from the sequenced genome, transcriptional expression [21] and experimental data [22], 36 putative sugar transporters were reannotated ( Additional file 1). Of them, 22 were with detectible transcripts and 5 were experimentally characterized. The 8 disaccharide transporters exhibited high homologues to the corresponding transporters in *S. cerevisiae*, but not the remaining 28 monosaccharide transporters for some of which were unique to *S. stipitis*, such as the arabinose-proton symporter (AUT) and xylose transporters (XUT), reflecting the capabilities of pentose utilization [23]. The newly annotated sugars transporters not only give us a sight into the molecular basis of sugar transport but also could be used as foundation for the identification and functional illumination of the various sugar transporters supposed to exist in *S. stipitis*. A map illustrating the central carbohydrate metabolism is provided (Figure 2). Upon entry into the cell, hexose (glucose, mannose, and galactose) are easily phosphorylated to enter the central metabolism while the pentose (xylose and arabinose) must pass through oxidoreductase reactions before phosphorylation. For instance, L-arabinose must go through four oxidoreductase steps via L-arabitol, L-xylulose, D-xylitol, and D-xylulose to enter pentose phosphate pathway (PPP). The additional steps in pentose converting pathways cause the loss of substrate carbon into the cell mass which may account for the less efficient of pentose than hexose for cell growth [23]. Except for the common lignocellulose-derived sugars (glucose, mannose, galactose, xylose, and arabinose), the novel sugar metabolic pathways such as cellobiose metabolism encoded by eight genes (*BGL1-7* and *SUN4*) and rhamnose metabolism encoded by four genes (*LRA1-4*) were also added to the model by extensive literature mining [23-25].

**Figure 3:**
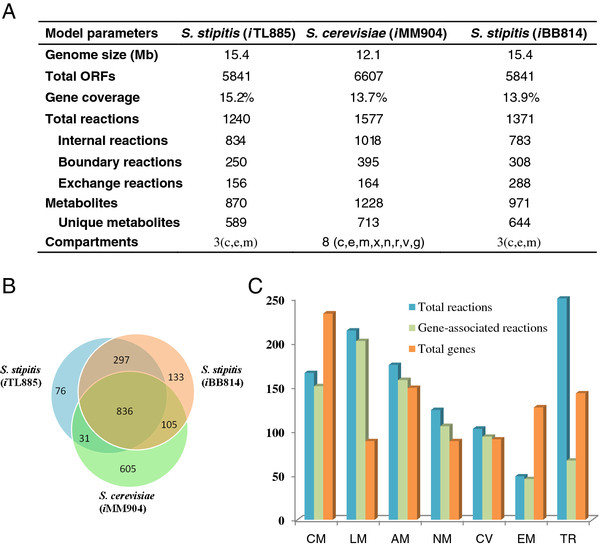
**Model characteristics of*i*TL885. A**) Model contents of two *S. stipitis* (*i*TL885 and *i*BB814) and one *S. cerevisiae* (*i*MM904). **B**) Venn diagram showing the number of total, shared and unique reactions in the three yeasts GSMMs. **C**) Statistics of the number of total reactions, gene-associated reaction and total genes in the seven cellular subsystems. Each reaction (except exchange reactions) in the model was assigned to a single subsystem. Abbreviations**:** CM (carbohydrate metabolism), LM (lipid metabolism), AM (amino acid metabolism), NM (nucleotide metabolism), CV (cofactors and vitamins metabolism), TR (transport reactions), and EM (energy metabolism).

The resulting genome-scale metabolic model *i*TL885 comprises 885 metabolic genes, 1240 reactions, and 870 metabolites. The 1240 metabolic reactions were distributed over 61 metabolic pathways and located into three cellular compartments. Compared with *i*MM904 (13.9%) and *i*BB814 (13.7%), the gene coverage of *i*TL885 is the highest, about 15.2% (Figure 3A). Altogether, there are 421 metabolites shared by the three models, and the unique metabolites were 589, 644, and 713 for *i*TL885, *i*BB814, and *i*MM904, respectively. The relatively small number of metabolites in *i*TL885 was caused by the excluding of tRNA-charging and dipeptide metabolism because of the presence of dead ends in these subsystems. For the reactions, 836 reactions are shared by the three models (Figure 3B), and the 76 unique reactions in *i*TL885 were mainly associated with the degradation of polysaccharide (chitin, xylan, mannan, and glycogen) and metabolism of glycerolipid and sphingolipid. In addition, nearly 92% of the metabolic reactions were associated with certain genes and the carbohydrate metabolism contained the largest percentage of metabolic genes, which was consistent with the physiological property of *S. stipitis* to use most of the sugars present in biomass substrates [26]. Meanwhile, this model captured 252 non-gene associated reactions, among which 183 were transport reactions (Figure 3C). The numerous transport reactions were included to represent the exchange of metabolites between different compartments although most of them didn’t have known gene associations due to the insufficient gene annotations or literature data. See Additional file 2 for the detailed description of the model structure.

### Gene essentiality study

**Figure 4:**
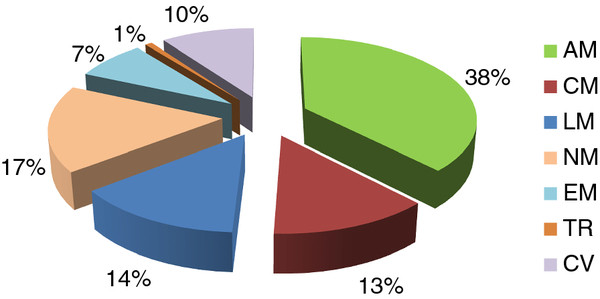
**Percentage of essential genes in each subsystems with glucose as carbon source.** The abbreviations of each subsystems are the same with that in Figure 3 .

The essentiality of each gene to cell growth was evaluated by removing each individual reaction from the stoichiometry matrix (see Method section). In the model *i*TL885, 130 genes (14.7% of the total genes) were predicted to be essential on a minimal medium with glucose as carbon source. This percentage was close to that of *S. cerevisiae* (12.9%) [27]. As illustrated in Figure 4, approximately 82% of the predicted essential genes were focused on amino acid metabolism (49 genes), nucleotide metabolism (22 genes), lipid metabolism (18 genes), and carbohydrate metabolism (17 genes), highlighting the significance of them to cell growth. Compared with the glucose medium, four additional essential genes were identified on the xylose medium. The four genes exclusively belonged to xylose metabolism were *XYL1* (PICST_89614), *XYL2* (PICST_86924), *PGI1* (PICST_84923), and *TAL1* (PICST_74289). Of them, *XYL1* (xylose reductase) and *XYL2* (xylitol dehydrogenase) encode the two initial enzymes of xylose metabolic pathway, which has been introduced to *S. cerevisiae* for the production of lignocellulosic ethanol [28]. The other two genes *PGI1* (glucose-6-phosphate isomerase) and *TAL1* (transaldolase) link the pentose phosphate pathway to glycolysis by the formation of key glycolysis intermediate D-fructose 6-phosphate (Figure 2). Many nonessential genes for cell growth could be explained by the existence of isoenzymes or alternative metabolic pathways. For example, the five ethanol dehydrogenase genes (*ADH1-5*) were predicted to be nonessential on the xylose medium. Actually, two independent ethanol synthesis pathways were identified. One is the common cytoplasmic ethanol pathway, the other is the mitochondrial pathway encoded by *ADH3* (PICST_88760), a mitochondrial alcohol dehydrogenase (absented in *i*BB814). However, the latter pathway functioned only when the cytoplasmic pathway was eliminated, which had been experimentally validated [29]. The list of essential genes was provided in Additional file 3.

### Physiological characteristic of ethanol fermentation

**Figure 5:**
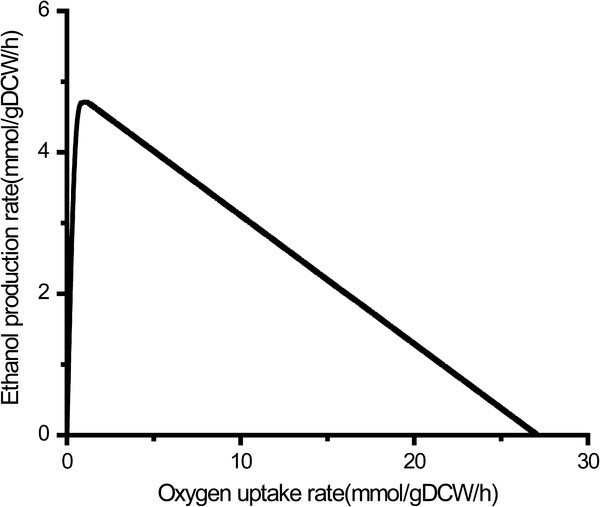
**Robustness analysis of oxygen uptake rate.** By fixing the xylose uptake rate and cell growth rate, the production of ethanol was maximized at each oxygen uptake rates.

Oxygen is a key factor that affects the production of ethanol in *S. stipitis*[14,30,31]. Here, the influence of oxygen on ethanol formation was studied by a computational robustness analysis with xylose as carbon source and ethanol synthesis as the objective function. The predicted result was illustrated in Figure 5. It was found that ethanol production rate increased sharply when oxygen uptake rate was at the range of 0.34 to 0.88 mmol/gDCW/h. The maximum ethanol production rate reached to 4.72 mmol/gDCW/h when oxygen consumption rate was 1.15 mmol/gDCW/h, in agreement with earlier experimental observation [14]. However, when oxygen consumption rate exceeded 1.15 mmol/gDCW/h, ethanol production rate began to decrease, and eventually to zero (oxygen consumption rate of 27.1 mmol/gDCW/h), due to the oxidation of ethanol in the presence of excessive oxygen [32]. The result indicates that ethanol production is strongly affected by the intracellular reduction-oxidation status. The secretion of ethanol occurs under oxygen-limited conditions and the maximum ethanol production could be achieved only under the relatively low oxygen condition. Besides, the produced ethanol can be reassimilated when oxygen is excessive.

**Figure 6:**
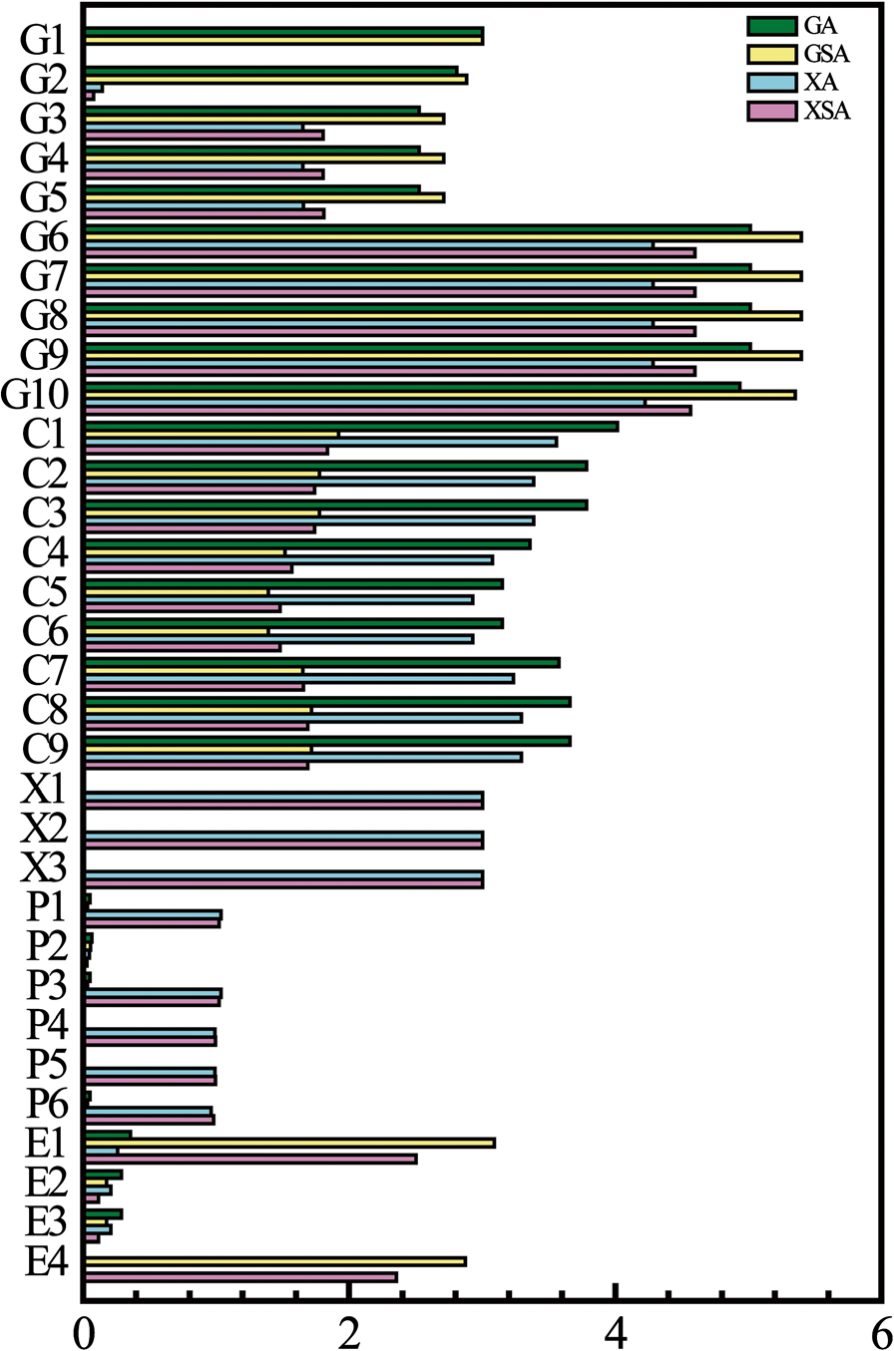
**Flux distributions of central metabolic reactions under four different growth conditions.** The length of each bar indicates the flux value of each reaction. Four conditions are GA (glucose aerobic), GSA (glucose semiaerobic), XA (xylose aerobic), and XSA (xylose semiaerobic). Briefly, G is for glycolysis, C for citrate cycle, X for xylose degradation, P for Pentose Phosphate Pathway, and E for ethanol synthesis. The detailed information of abbreviated reactions is available in Additional file 3 .

More specifically, the distributions of carbon flux under different oxygen levels (aerobic and semiaerobic) were investigated by flux balance analysis (FBA) (Figure 6). It was found that (i) the glycolytic flux wasn’t obviously affected by the oxygen levels by either glucose or xylose, but the flux channelled into tricarboxylic acid cycle (TCA cycle) increased about twofold when the cells were shifted from semiaerobic to aerobic, which were consistent with the reported results that genes involved in TCA cycle were downregulated as oxygen availability decreased [33]; (ii) at the node of pyruvate, the flux to acetaldehyde (leading to fermentation) increased about tenfold with the decreased oxygen level, which was in accordance with the experiment that the expression of key fermentative genes (*PDC*, *ALD* and *ADH*) for ethanol production were greatly improved under the oxygen-limited condition [34]. Meanwhile, the effects of different carbon sources (xylose and glucose) on the flux distribution of central metabolism were also analysed. Xylose greatly increased the carbon flux channelled into the PPP. The sufficient PPP activity in *S. stipitis* has spurred numerous studies on the overexpression of non-oxidative PPP genes (*TAL1*, *RPE1, RKI1*) to improve the utilization of xylose in the recombinant *S. cerevisiae*[8] as well as in *S. stipitis*[35]. However, the metabolic fluxes through TCA cycle do not change significantly with the two carbon sources, indicating the expression of the enzymes-coding genes remained constant [21].

### Ethanol production from xylose

Previous study of xylose utilization in *S. stipitis* was mainly focused on the illustration of transport mechanism and metabolic pathway [36,37]. In the model *i*TL885, seven putative high-affinity xylose transporters (XUT1-7) were annotated, and one of them (XUT1) has been biochemically characterized from *S. stipitis*[38]. However, the low-affinity xylose proton symport systems could not be found just by gene annotation. So, kinetics experiment was necessary to determine the low-affinity xylose transporters. Four Sut proteins (SUT1-4) with higher affinity for glucose than xylose had been characterized in this way, indicating this low-affinity system is shared by glucose and xylose [16,38]. As a result, the influx of xylose could be attributed to the cooperation of the two xylose transport systems. Besides, sugar sensors (SNF3, RGT2, etc.) were also identified in *S. stipitis* genome, which ensures the quick cellular response to the change of xylose in the environment, as the transcriptional data showed that the transcription of RGT2 with xylose could increase 65-fold [21].

The assimilated xylose can be metabolised via two catabolic routes: xylulokinase pathway and D-arabinose utilization pathway (Figure 2). Xylulokinase pathway is a well characterized pathway including three reactions encoded by three genes *XYL1* (PICST_89614), *XYL2* (PICST_86924), and *XKS* (PICST_68734), respectively [39]. Based on our simulation, this pathway is thought to be the only route redirecting carbon flux from xylose to PPP in the wild-type cell. The D-arabinose utilization pathway consists of three biochemical reactions catalyzed by D-arabinitol dehydrogenase (*AAD1*), D-ribulose reductase (*ARD2*) and D-ribulokinase (*RKS1*), which was found due to the fact that xylose still can be metabolised in the XKS disruption strain [40]. This pathway was also responsible for the degradation of D-arabinose, as illustrated in Figure 2.

**Figure 7:**
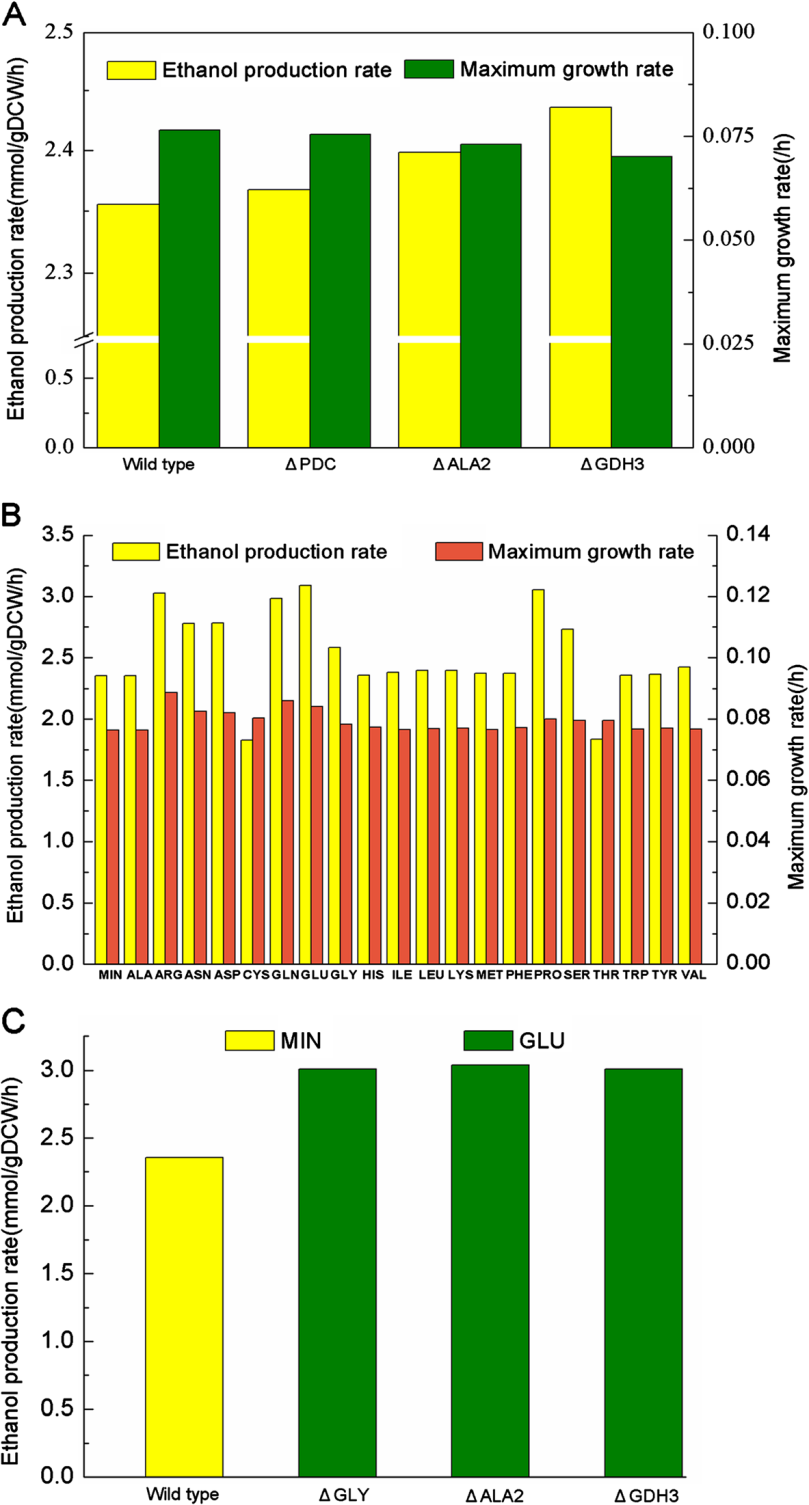
**Predicted ethanol production rates under different conditions. A**) Ethanol production rate and maximum growth rate for the wild type cell and three in silico mutant strains (deletion of *GLY1* and *GLY2, ALA2*, *GDH3* respectively) on minimal medium. **B**) The maximum growth rate and ethanol production rate on minimal medium and minimal medium with one of the 20 amino acids. **C**) The predicted ethanol production rate of the gene deletion strains on minimal medium with glutamate (GLU) compared to the wild type cell on minimal medium (MIN).

Efficient production of xylose-derived ethanol is of particular interest for *S. stipitis*[41,42]. To gain a higher productivity of ethanol, three promising gene knockout strategies were identified by OptKnock algorithm (Figure 7A). The first one was a straightforward strategy to increase the available pool of the acetaldehyde precursor by knocking out of two threonine aldolase (GLY) genes (PICST_7413 and PICST_71203), which blocked the flux from acetaldehyde to threonine. But this method showed only a slight increase of ethanol production rate. The second one was to enhance the mitochondrial pyruvate pool through the deletion of alanine transaminase gene *ALA2* (PICST_70108), which led to a considerable increase of ethanol accumulation rate. The third effective strategy was to augment the supply of NADPH by deleting the NADP-dependent glutamate dehydrogenase gene *GDH3* (PICST_82969). This optimization approach not only increased ethanol production rate about 3.4%, but had a negligible influence on cell growth (with the maximum growth rate remain at 0.07 mmol/gDCW/h).

The optimization of organic nitrogen sources had been reported to be able to enhance the bioconversion of xylose [43], thus the influence of specific amino acids on the production of ethanol were computationally investigated (Figure 7B). The addition of 17 of the 20 amino acids could improve both cell growth and ethanol production. Of those positive additions, the addition of glutamate had the most significantly impact, leading to the ethanol production rate increased by 27.7% compared to the control. Combined the above two strategies, i.e. gene deletion and glutamate addition, the production rate of ethanol was predicted to increase by over 20.0% for all the deletion strains, among which the addition of glutamate to the *ALA2* deletion strain could improve ethanol production rate up to 1.29 fold of the control (Figure 7C). The results suggested that future work for the optimization of ethanol fermentation in *S. stipitis* should also consider the availability of nutrients aside from carbon source, which perhaps will be more beneficial as the cost-effective media often contain complex mixtures of nutrient derived from natural substrates.

## Conclusions

In summary, an *in silico* model named *i*TL885 was developed representing a comprehensive knowledge of the metabolism of *S. stipitis*. Compared with the reported *i*BB814, *i*TL885 possessed a higher gene coverage rate in despite with a slightly smaller model size. Model-driven study of the gene essentiality validated the role of key metabolic genes in xylose metabolism and ethanol synthesis. Nevertheless, a further large-scale gene knockout study of *S. stipitis* is necessary for a better elucidation of its genotype-phenotype relationships. Robustness analysis demonstrated the profile of ethanol formation and FBA pointed out the impacts of oxygenation and carbon sources on the flux distribution of central metabolism. In light of the fermentation characteristic, we suggested the well-controlled oxygen concentration to achieve the maximum production of ethanol. The investigation of xylose utilization from the perspectives of sugar transport and metabolism partly accounts for the high efficient bioconversion of xylose in *S. stipitis*. For the overproduction of ethanol from xylose, candidate knockout targets were identified and the effects of amino acids addition were simulated, which proves that GSMM is capable of designing optimal culture conditions and metabolic engineering strategies. Therefore future work for *S. stipitis* can be focused on the experimental testing of strain design hypothesis generated by the computational analysis.

## Methods

### Model reconstruction and refinement

The availability of whole genome of *P. stipitis*[44] enables us to carry out the model reconstruction following the general workflow of GSMM reconstruction described before [45,46]. Firstly, the sequenced genome data of *P. stipitis* CSB 6054 was downloaded from UniProt database [47]. Then, the functional annotation of genes was performed in two different ways. BLAST was used to conduct the sequence homology search of *S. stipitis* with two yeasts (*S. cerevisiae* and *P. pastoris*) and one fungus (*A. niger*). The thresholds of the bidirectional BLAST (BLASTp) for a functional sequence were set to have an e-value less than 1 × 10^-30^, an amino acid sequence identity above 40%, and a matching length at least 70% of the query sequence. To obtain a original reactions list, also called in-house model, GSMMs of *S. cerevisiae i*MM904 [27], *P. pastoris i*PP668 [48] and *A. niger i*MA871[49] were selected as template frameworks to map the assigned genes. On the other hand, KAAS [50] was used for the functional annotation of all the query amino acid sequences. With the specific KO identifiers or Enzyme Commission (EC) numbers, particular reactions were selected from the KEGG reaction database which was then integrated to the BLAST results. Special attention was paid on the most significant components of genome-scale network, the gene-protein-reaction (GPR) associations which details the relationships between genes, proteins and reactions using the Boolean logical representations (AND and OR operators). The various isoenzyms or enzyme complexes were identified with the help of KEGG Modules [51] and assignments of homologous genes in *S. cerevisiae*. As a result, a draft model was developed and used as a start point for subsequent network refinements. With the biochemical information acquired from public databases such as KEGG [51], MetaCyc [52], BRENDA [53], and TCDB [54], manual revisions including deletion of error reactions, addition of organism-specific information, checking of mass-charge balance and filling of metabolic gaps were conducted one by one. GapFind and gapFilling in the Constraint-Based Reconsruction and Analysis (COBRA) toolbox were performed to identify and bridge the gaps in the current version of model so as to match the genotype and phenotype [55]. A detailed model structure was accomplished when a biomass formation reaction and certain exchange reactions were added to the network. Biomass equation is an artificial linear combination of all the known biomass constituents and their defined proportions. Exchange reactions describe the uptake of nutrients from the medium and the secretion of specific metabolites to the extracellular environment, thus defining the systems boundaries.

### Modelling technique

All computational simulations were performed using COBRA toolbox [56] on Matlab (The MathWorks Inc., Natick, MA) with GLPK as the linear optimization solver. FBA was used as the main algorithm for network modelling and analysis. The mathematical formulation and numerous applications of FBA have been reviewed [57]. Briefly, FBA is an effective tool for the prediction of the maximal cell growth and metabolite production when given certain constraints. The output of FBA is an optimal flux distribution for each reaction in the model and a maximum value for the objective function. Generally, the biomass equation is set as the objective function for the simulation of optimal growth and other model evaluations such as essentiality study.

All the simulations were performed on a minimal medium with limited carbon source. The uptake rates of ammonia, sulphate, phosphate, sodium, kalium, and ferrite were unconstrained with the lower and upper flux bounds of −1000 and 1000 mmol/gDW/h respectively. Xylose or glucose was set as sole carbon source with a uptake rate of 3 mmol/gDCW/h [14]. The robustness analysis of oxygen uptake was performed by fixing the xylose uptake rate and cell growth rate (0.01 h^-1^) to predict the maximum ethanol production rate at each controlled oxygen uptake rate. The aerobic condition was simulated by uncostraining the oxygen uptake rate, and the semiaerobic condition by constraining the exchange reaction of oxygen to −5 mmol/gDW/h. To modelling the amino acids addition, each of amino acid was constrained to have a maximum consumption rate of 0.1 mmol/gDCW/h.

### Gene deletion simulations

The computational single gene deletion was conducted to predict the important genes for the synthesis of building blocks of cellular biomass [58]. For a given gene, the *in silico* knockout was performed by constraining the flux value of its corresponding reaction to zero when maximizing the growth rate. This method also works for the complex GPRs such as isoenzymes and multiplex enzymes. If the maximum growth rate of the gene knockout strain is less than 1 x 10^-6^ of the wild type, the deleted gene is defined as essential. Otherwise, it’s a nonessential gene. The OptKnock algorithm in COBRA toolbox identifies candidate genes that lead to the overproduction of desired metabolite [59,60]. With ethanol as the target product, OptKonck was applied to discover target gene(s) knockouts. The scope of OptKonck was constrained to the nonessential genes in central metabolism (glycolysis, TCA, and PPP) and amino acid metabolism.

## Abbreviations

GSMM: Genome-scale Metabolic Model; BLAST: Basic Local Alignment Search Tool; KO: KEGG ORTHOLOGY; KEGG: Automatic Annotation Server (KAAS); COBRA: Constraint-Based Reconsruction and Analysis; PPP: Pentose Phosphate Pathway; TCA cycle: Tricarboxylic Acid Cycle; GPR: Gene-Protein-Reaction; XYL1: Xylose reductase; XYL2: Xylitol dehydrogenase; XKS: D-xylulokinase; PGI1: Glucose-6-phosphate isomerise; TAL1: Transaldolase; ADH: Ethanol Dehydrogenase; PDC: Pyruvate Decarboxylase; ALD: Aldehyde Dehydrogenase; RPE1: D-ribulose-5-phosphate 3- epimerase; RKI1: Ribose-5-phosphate Ketol-isomerase; SNF3: High-affinity glucose transporter; RGT2: Glucose sensor; XUT: Xylose Transporters; SUT: Hexose Transporter; AAD1: D-arabinitol Dehydrogenase; ARD2: D-ribulose reductase; RKS1: D-ribulokinase; GLY: Threonine aldolase; ALA2: Alanine transaminase; GDH3: NADP-dependent glutamate dehydrogenase.

## Competing interests

The authors declare that they have no competing interests.

## Authors’ contributions

TL constructed the model and drafted the manuscript. WZ provided suggestions for model analysis and simulations. LML designed the experiments and JC revised the manuscript. All authors read and approved the final manuscript.

## Supplementary Material

Additional file 1 **Genome reannotation.** This Excel workbook contains the detailed gene annotation results by BLAST and KAAS, as well as the 36 reannotated sugar transporters. Click here for file

Additional file 2 **Model structure.** This Excel workbook contains detailed information of *S. stipitis i*TL885, including all the reactions, metabolites, and GPRs. Click here for file

Additional file 3 **Essential genes.** This Excel workbook is a list of the predicted essential genes with glucose as sole carbon source and the original reactions corresponding to the abbreviations in Figure 6. Click here for file
